# Acute effect of photobiomodulation therapy in quadriceps strength, fatigue and pain of patients with chronic kidney failure: a randomized, double-blind, placebo controlled crossover clinical trial

**DOI:** 10.1007/s10103-026-04886-5

**Published:** 2026-05-23

**Authors:** Ana Paula Oliveira Barbosa, Lidiane Martins Santos, Amanda Marques Catelli, Degiane Rocha da Rosa, João Carlos Goldani, Graciele Sbruzzi Graciele Sbruzzi, Rodrigo Della Mea Plentz, Jociane Schardong

**Affiliations:** 1 Universidade Federal de Ciências da Saúde de Porto Alegre (UFCSPA), Porto Alegre, Brazil; 2 Santa Casa de Porto Alegre (SCPA), Porto Alegre, Brazil; 3 Universidade Federal do Rio Grande do Sul (UFRGS), Porto Alegre, Brazil

**Keywords:** Kidney failure, chronic, Renal dialysis, Low-level light therapy, Phototherapy, Randomized controlled trial

## Abstract

Patients with chronic kidney failure (CKF) present changes in physical capacity and low exercise tolerance. Photobiomodulation therapy (PBMT) has shown an ergogenic effect on exercise in studies with different populations and may be an adjuvant treatment in rehabilitation programs for CKF patients. This study aimed to evaluate the acute effects of different doses of PBMT on quadriceps strength, fatigue and muscle pain in CKF patients. This study is a randomized, placebo-controlled, double-blind, crossover clinical trial. Adult patients with CKF received four PBMT applications with different energy doses (30J, 60J, 90J, or placebo), administered in a random order. A one-week interval was maintained between each dose, and an 830nm laser was applied to six points of the quadriceps muscle. Immediately after PBMT, maximal isometric quadriceps muscle strength was assessed by dynamometry. Before the intervention and after the assessment of muscle strength, muscle fatigue (Borg rating of perceived exertion scale and blood lactate) and pain perception in the lower limbs (visual analogue scale) were measured. Fifteen patients were randomized and fourteen completed the study. There was no significant difference between the doses tested for maximal isometric muscle strength in the right (p=0.053) and left (p=0.509) quadriceps. The perception of fatigue assessed by the modified Borg scale was not altered (p=0.703) nor were blood lactate levels, regardless of the PBMT dose and measurement time (t0: p=0.126; t3: p=0.667; t6: p=0.700). There was no change in pain perception after quadriceps irradiation for any of the PBMT doses tested (p=0.566). A single application of PBMT with a dose of 30J, 60J, or 90J did not alter maximal isometric quadriceps muscle strength, fatigue, or muscle pain perception in the lower limbs of CKF patients on HD.

## Introduction

Chronic kidney disease (CKD) is a condition that affects the health of millions of people around the world, with an estimated global prevalence of 9.5% of the population [1]. CKD is defined by parenchymal damage and/or decreased renal function for a period longer than three months [2]. In the most severe phase of the disease known as chronic kidney failure (CKF), patients are candidates for kidney transplantation and/or life-sustaining renal replacement therapy, such as hemodialysis (HD) [3, 4].

Organic dysfunction resulting from kidney failure and the accumulation of uremic toxins is associated with cardiovascular, musculoskeletal, inflammatory, endocrine, neurological and respiratory disorders, among others [5]. Patients with CKD have a protein synthesis imbalance caused by metabolic acidosis, resistance to insulin and insulin-like growth factor type 1 (IGF-1), hormonal changes, cytokines, mitochondrial respiratory chain dysfunction, inflammatory processes, diminished appetite and increased malnutrition [6–9]. Thus, they have low total lean body mass density and loss of muscle fibers, mainly type II [7].

Sarcopenia is characterized by reduced muscle strength associated with decreased mass and/or reduced muscle function and affects approximately 20% of kidney patients undergoing HD [10]. Sarcopenia and chronic fatigue due to exertion are related to reduced exercise capacity and tolerance, decreased quality of life and increased mortality in CKF patients [11]. Therefore, lifestyle changes such as the adoption of physical exercise are an excellent non-drug intervention to mitigate the effects of uremic syndrome [11–13], thus reducing the risk of disease progression and severity [14].

Studies have explored photobiomodulation therapy (PBMT) due to analgesic, anti-inflammatory, and regenerative effects [15–17], as well as an ergogenic strategy for exercise [18]. PBMT refers to the use of photons as non-thermal, non-ionizing radiation to alter biological activity for therapeutic purposes. Increased expression of cytochrome c-oxidase (CCO) photoreceptors in the mitochondrial respiratory chain, stimulated by PBMT, leads to an increase in adenosine triphosphate (ATP) production, in photodissociation of nitric oxide (NO) [19], in promoting lactate removal [20] and reactive oxygen species (ROS) level modulation [21]. These effects improve mitochondrial activity providing greater cellular energy, reducing oxidative stress and favoring microcirculation [19–21]. Furthermore, PBMT may stimulate the release of calcium (Ca²⁺) from the sarcoplasmic reticulum and enhance Ca²⁺ influx through membrane channels, optimizing muscle contraction and performance [22].

Patients with CKF and long-term HD have a deregulated and compromised mitochondrial respiratory system, mainly in the expression of proteins of the subunit I e IV of complex IV of the oxidative phosphorylation system; and these findings were associated with increased oxidative stress and the production of ROS [19]. In addition, these patients typically present low levels of physical activity and intolerance to overload, therefore, PBMT can be a strategy to improve muscle function, making exercise easier. A previous study on this population observed positive results when evaluating the acute effect of PBMT on handgrip strength [19].

Despite the compelling theoretical rationale supporting PBMT for enhancement of muscle function, empirical evidence demonstrates substantial heterogeneity in photobiomodulation efficacy across different populations and target tissues. Specifically, the magnitude of photobiomodulation effects varies according to: (1) the anatomical type and mass of the irradiated muscle group, with smaller muscles such as intrinsic hand muscles potentially exhibiting a greater response than large muscle groups; (2) subcutaneous tissue thickness and adipose tissue distribution, which attenuate light penetration; (3) the specific optical dose and wavelength utilized, with effects often demonstrating non-linear dose–response relationships; and (4) the underlying pathophysiological context, with disease-specific mitochondrial dysfunction potentially modifying therapeutic responsiveness. Whilst Macagnan et al. [19]. demonstrated acute beneficial effects of PBMT on handgrip strength in patients with CKF, other randomized controlled trials in populations with heart failure [23] or following coronary artery bypass grafting [24] reported divergent results, with no significant improvement in functional outcomes. Consequently, the extrapolation of positive PBMT effects demonstrated in small, superficial muscle groups to the large quadriceps femoris muscle in patients with chronic kidney failure cannot be assumed and requires direct empirical investigation.

To the best of our knowledge, no previous study has evaluated the acute effects of PBMT on the lower limbs of patients with CKF undergoing hemodialysis. Furthermore, the optimal dose of PBMT for this muscle group is unknown. We hypothesized that a single application of PBMT would increase quadriceps muscle strength during exercise and reduce or prevent worsening muscle fatigue and pain in these patients after effort. Thus, this study aimed to evaluate the acute effect of different doses of PBMT on isometric quadriceps muscle strength, fatigue and muscle pain in patients with CKF on HD.

## Patients and methods

### Design

This study is a randomized, double-blind, placebo-controlled crossover clinical trial, conducted in accordance with the Consolidated Standards of Reporting Trials (CONSORT) recommendations [25, 26]. Isometric quadriceps muscle strength was considered the primary outcome of the study. Fatigue and muscle pain perception were considered secondary outcomes.

### Human ethics and consent to participate declarations

The project was reviewed and approved by the Human Research Ethics Committees of Santa Casa de Porto Alegre (SCPA) hospital, under CAAE number 59705722.1.0000.5335 and approval number 5.531.413. Furthermore, it was registered in ClinicalTrials.gov prior to participant enrolment (NCT05881772; May 21, 2023). All primary and secondary study outcomes were pre-specified in the protocol registration and reported in this manuscript. The evaluations and procedures were performed at the HD outpatient center at Policlinica Santa Clara of SCPA hospital, between June and August 2023, in accordance with the ethical standards of the Declaration of *Helsinki*, revised in 2013 in Brazil. The volunteers that fulfilled the eligibility criteria signed the free and informed consent form to participate prior to any procedure.

### Eligibility criteria

Patients with CKF on HD for a period of at least three months, of both sexes, aged between 18 and 80 years, with adequate urea clearance [urea reduction ratio (URR) ≥ 65%], and a weekly dialysis frequency of three times per week were included in the study. Exclusion criteria were: inability to understand commands to perform the evaluations; epidermal lesions at the site of the PBMT application; stroke sequelae; recent acute myocardial infarction (two months); uncontrolled hypertension (systolic pressure > 230 mmHg and diastolic pressure > 120 mmHg); IV grade heart failure according to the New York Heart Association or decompensated; unstable angina; deep venous thrombosis in the lower limbs; incapacitating osteoarticular or musculoskeletal disease preventing the performance of physical evaluation tests; uncontrolled diabetes (glycemia>300 mg/dL); febrile state and/or infectious disease, systemic lupus erythematosus; and cancer.

### Procedures

All the patients from HD outpatient center were invited to participate in the study. Those who showed interest had their electronic medical records consulted to verify the eligibility criteria. Identification, demographic and anthropometric data, risk factors and cause that led to CKF were collected from patients included in the study. After enrollment, patients were randomized to the order in which they would receive the four doses of PBMT. The assessment and intervention procedures were carried out in a multidisciplinary office, at the HD outpatient center, in a quiet environment without external interruptions.

### Randomization and blindness

The patients received four doses of PBMT, with a one-week interval between them, and according to the individual randomization order. Randomization was performed by a researcher external to the study, using the www.random.org website. The dose was revealed to the intervention therapist only before the application of the PBMT to ensure concealment of the allocation. Participants and the outcome evaluator were blinded to the therapeutic dose.

Participants wore protective eyewear with dark lenses and headphones playing music during PBMT applications to prevent visual or auditory cues that could reveal the intervention. To eliminate the perception of differences in exposure time between doses, the cluster was maintained in contact with the skin at each application site for a total of one minute, even when the irradiation time required to deliver the dose was shorter (chart 1); during the remaining time, the device remained switched off. Irradiation with PBMT did not generate any sensation. Patients only perceived the tactile stimulus associated with the device being in contact with the skin during application.

**Chart 1:** Parameters for TPBMT application

Parameter	
Wavelength	830 nm
Emission mode	Continuous
Output power (cluster)	800 mW
Output power (per diode)	200 mW
Number of diodes (cluster probe)	4
Total area of the cluster	0.273 cm²
Spot size (per diode)	0.159 cm²
Power density (cluster)	5.03 W/cm²
Power density (per diode)	1.26 W/cm²
Dose (cluster)	30/60/90 J
Dose (per diode)	7.5/15/22.5 J
Density (cluster)	188.7/377.4/566 J/cm²
Density (per diode)	47.2/94.3/141.5 J/cm²
Time of application (per site)	20/40/60s
Number of sites (per leg)	6
Total Dose (per leg)	180/360/540 J

### Evaluations

**Fig. 1:**
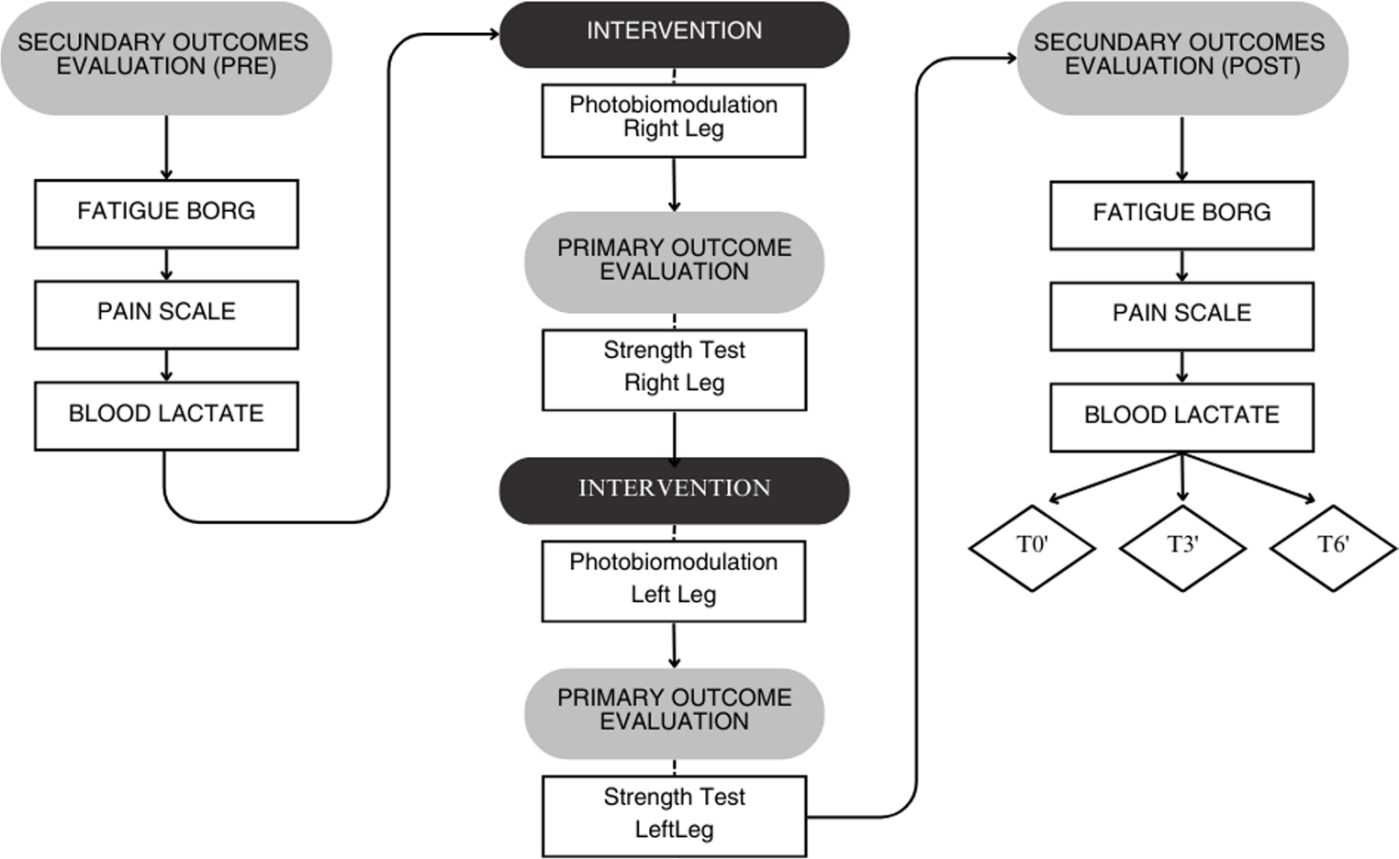
Procedure of data collection

All assessment procedures were performed prior to the second weekly hemodialysis session over four weeks. The PBMT doses were administered at one-week intervals to avoid residual effects from the previous dose, consistent with washout periods commonly adopted in crossover PBMT studies [19, 27, 28]. Patients were instructed not to perform any effort or physical exercises prior to the assessment and to wear comfortable clothing on the day of the tests. The assessments were performed before irradiation of the quadriceps with PBMT in the following order: (1) Perceived exertion; (2) Measurement of blood lactate; (3) Assessment of muscle pain in the lower limbs. After the PBMT application and the subsequent performance of the maximal isometric quadriceps strength test, the following assessments were repeated: (4) Perceived exertion; (5) Measurement of blood lactate (times 0, 3 and 6 min); (6) Assessment of muscle pain in the lower limbs (Fig. 1). All assessments were conducted by trained researchers who were blinded to the doses of PBMT applied.

a) Maximal Isometric Quadriceps Strength Evaluation

**Fig. 2:**
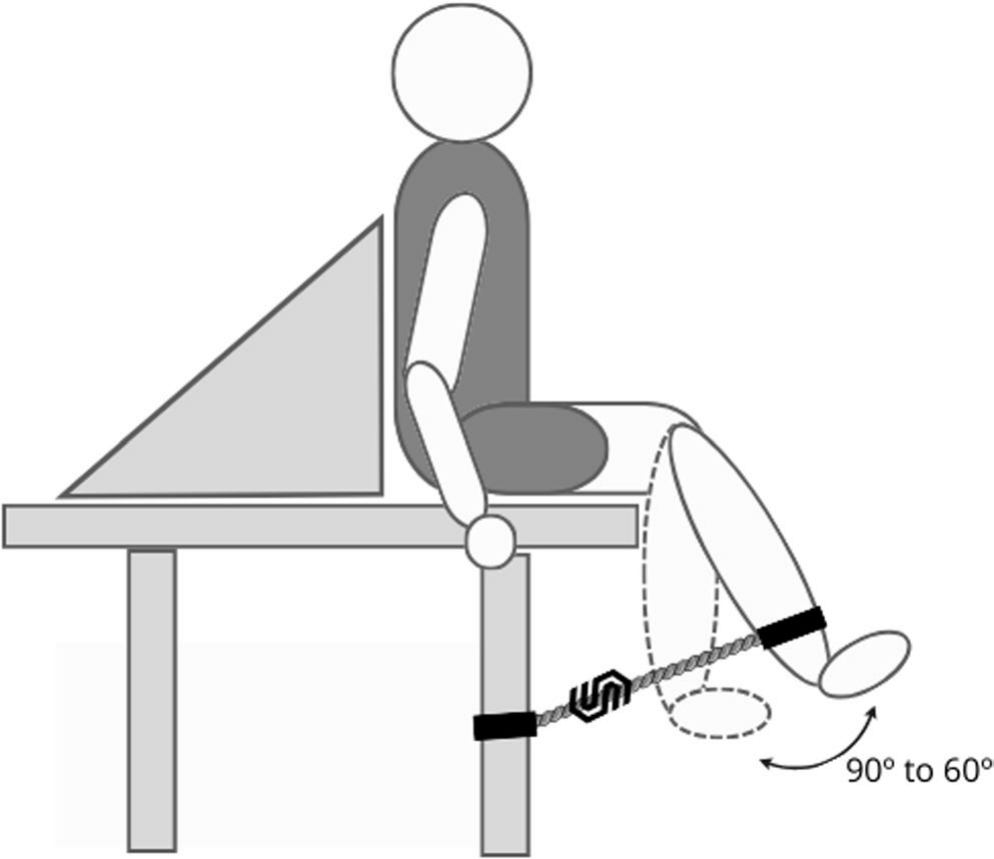
Assessment of isometric quadriceps muscle strength by load cell

The maximal isometric quadriceps strength was measured by dynamometry using a load cell (SDS 1000, traction mode, nominal capacity of 200 kg, 0.1 kg resolution). It was calibrated and connected to a data acquisition system (Miotool, model 400 USB, Miotec, Porto Alegre/RS, Brazil). The patient was positioned in a sitting position on a litter with an adapted back support. The initial position was an angle of 90 degrees of hip and knee flexion. The participant was asked to perform and maintain knee extension at a 60º angle (Fig. 2). As in a previous study, the load cell position was fixed using a velcro ankle strap to guarantee a perpendicular alignment of the sensor [29]. For the patient’s angle position, an acrylic goniometer (ProFisiomed, Porto Alegre, RS, Brazil) with a one-degree resolution was used.

The patient was instructed to perform and sustain the maximal isometric force generated by the quadriceps for five seconds while receiving verbal encouragement through standardized phrases [29, 30]. A minimum number of three and a maximum of five measurements were taken, with a two-minute rest interval between them. Only standardized measurements were considered valid, excluding those with a value greater or less than 10%. The force peak generated was considered for analysis. Measurements were recorded using the MiotecSuite 1.0 software and analyzed using the Miograph program and expressed in kgf units.

Maximal isometric right quadriceps strength was assessed immediately after PBMT irradiation of the patient’s right lower limb, approximately 45–60 s post-application. After completion of the right-limb measurements, PBMT was applied to the contralateral limb, followed by the same strength assessment. Details of the PBMT intervention are described below.

b) Muscle Fatigue Evaluation

Muscle fatigue was measured using the Borg Rating of Perceived Exertion Scale and blood lactate levels. The patient was asked about the “tiredness of his legs” and instructed to rate it, where 0 indicates no effort or fatigue and 10 indicates maximal effort [31].

Lactate levels (mmol/L) were measured through a drop of blood on a BM-Lactate reagent strip (Roche Diagnostics GmbH, Mannheim, Germany), which was then introduced into the Accutrend^®^ Lactate meter (Roche Diagnostics GmbH, Mannheim, Germany). To collect capillary blood, automatic self-lancets (G-Tech^®^ − 28G needle) were used at the tip of the index finger. Lactate was measured before PBMT application (baseline), immediately after the maximal isometric quadriceps strength evaluation of both legs (time 0 - t0), three (t3) and six (t6) minutes later [23].

c) Muscle Pain Evaluation

Lower limb muscle pain was measured using the visual analogue scale (VAS), where point 0 (zero) represents no pain and point 10 (ten) the worst possible pain. The patient was asked about the “muscle pain in their legs” and instructed to rate it using the scale [32].

### Intervention

PBMT was applied using a low-intensity infrared cluster laser with four diodes, 830 nm wavelength (HTM^®^, model Fluence MAXX, São Paulo, Brazil). Detailed parameters can be seen in chart C. All patients received four doses of laser radiation, with a one-week interval between each dose: 30 J (180 J per leg), 60 J (360 J per leg), 90 J (540 J per leg) or placebo. The doses were determined based on clinical and scientific recommendations for the use of PBMT for muscle performance [33], a meta-analysis [18], and previous studies [19, 34–36], considering the therapeutic window of 60 to 300 J for large muscle groups. Similarly, the PBMT application was performed immediately before the activity (strength test) for the expected acute effects, as previously described. The order of each dose application was determined by randomization. The placebo treatment was carried out with the equipment turned off.

For the application, the patient was maintained in the same sitting position as during the evaluations, and the treatment was performed with the probe fixed in direct contact with the skin at a 90° angle, using continuous emission mode. The therapy was applied to the quadriceps muscle, bilaterally. Two points were irradiated in the distal region of the vastus medialis, two points in the distal region of the vastus lateralis and two points in the central region of the rectus femoris, like a previous study [37]. A map of the application points was drawn after the first application with PBMT to ensure that the same locations were irradiated in the subsequent applications. All PBMT interventions were performed by the same therapist and in accordance with the previously described blinding procedures.

### Statistical analysis

The sample size calculation estimated 10 patients, and it was based on a previous study [37] that evaluated the acute effect of PBMT on maximal quadriceps isometric strength in patients with chronic obstructive pulmonary disease (COPD). The sample calculation considered a difference of 17.49 Nm for maximal voluntary isometric contraction, a standard deviation of 11.02, a power of 80% and an alpha error of 5%. Considering potential losses, the final sample size was increased to 15 patients.

Categorical variables were presented as absolute and relative frequencies, and quantitative variables as means and standard deviations or medians and interquartile ranges (IQR; P25–P75), as appropriate. Normality was assessed using the Shapiro–Wilk test. The peak strength value was used for the analysis of maximal isometric quadriceps strength, whereas the mean change (Δ = mean post − mean baseline) was used for fatigue (Borg scale) and muscle pain (VAS). A repeated-measures ANOVA was employed to assess a possible learning effect across the experimental sessions and to compare outcomes across PBMT doses. For blood lactate concentrations, a two-way repeated-measures ANOVA was performed including the factors dose (placebo, 30 J, 60 J, and 90 J) and time (baseline, t0, t3, and t6), and the dose × time interaction was also evaluated.

Lactate area-under-the-curve (AUC) was calculated to quantify the overall lactate exposure across the time-concentration curve during the recovery period. The AUC was estimated using the trapezoidal method based on lactate concentrations measured at baseline, immediately post-exercise (t0), and during recovery at 3 (t3) and 6 min (t6). For each pair of consecutive time points, the area was calculated as the mean of the two lactate concentrations multiplied by the corresponding time interval. The total AUC was obtained by adding up the areas of the trapezoids formed between baseline–t0, t0–t3, and t3–t6. The resulting values were expressed in mmol·min/L and compared between experimental conditions using repeated-measures analysis.

The effect size (Cohen’s *dz*) was calculated for each PBMT dose compared with placebo, as the mean of the paired within-subject differences divided by the standard deviation of those differences. The overall effect of time in the repeated-measures analysis was expressed using partial eta squared (η²p). The significance level was set at 0.05. Per-protocol analyses were conducted to assess the efficacy of PBMT among participants who completed all experimental sessions. Statistical analyses were performed using SPSS software (IBM SPSS Statistics for Windows, Version 25.0, Armonk, NY: IBM Corp.). G*Power software (version 3.1) was used to determine the sample size and to perform the post hoc statistical power analysis.

## Results

**Fig. 3:**
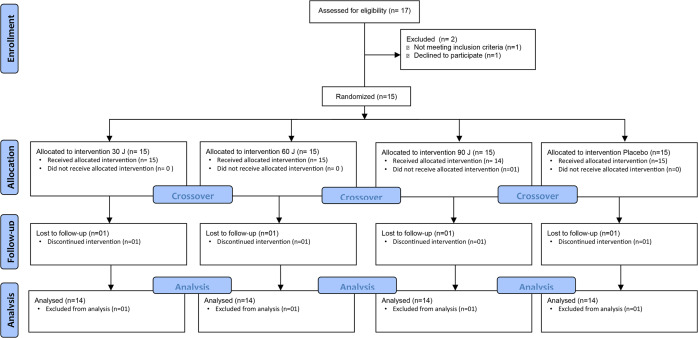
CONSORT Flow diagram of participants

The flowchart for patient selection and inclusion in the study is shown in Fig. 3. Seventeen CKF patients on HD were assessed for eligibility, fifteen were randomized, and fourteen were included in the analysis. Demographic, anthropometric and clinical characteristics of the patients at baseline of the study are summarized in Table 1.

**Table 1:** Characteristics of patients admitted to the study

	*n* = 14
Age (years)^a^	57.4 ± 15.2
Sex (male)^c^	9 (64.3)
Dry weight (kg)^a^	66.2 ± 16.9
Wet weight (kg)^a^	69.6 ± 18
Height (m)^a^	1.7 ± 0.1
BMI (kg/m^2^)^a^	23.6 ± 3.7
HD time (months)^b^	25.7 (17.7–59.4)
URR (%)^a^	72.6 ± 5.0
Primary disease^c^	
- Diabetes mellitus	4 (28.6)
- Polycystic kidney disease	2 (14.3)
- COVID-19	1 (7.1)
- NSAIDs induced nephrotoxicity	1 (7.1)
- Solitary kidney	1 (7.1)
- Hemolytic-Uremic Syndrome	1 (7.1)
- Unknown	4 (28.6)
Risk factors^c^	
- Ex-smoker	5 (35.7)
- Hypertension	11 (78.6)
- Sedentary lifestyle	11 (78.6)
- Diabetes mellitus	3 (21.4)
- Heart disease	4 (28.6)
- Peripheral vascular disease	1 (7.1)

Data are expressed as: a: mean ± standard deviation; b: median and interquartile range (P25-P75); or c: frequency and (percentage prevalence)

*M* male, *BMI* body mass index, *HD* hemodialysis, *URR* urea reduction rate, *NSAIDs* non-steroidal anti-inflammatory drugs, *FH* family history

No learning effect was observed during the muscle strength assessments. This is shown in Table 2, where the mean values of maximal isometric quadriceps strength obtained after PBMT application are compared across the four irradiation sessions, regardless of the dose used.

**Table 2:** Maximal isometric quadriceps strength in the first, second, third and fourth PBMT session, regardless of dose

	1 st session	2nd session	3rd session	4th session	*p*-value
Right limb strength (kgf)	26.5 ± 7.9	26.9 ± 6.7	28.4 ± 8.7	27.6 ± 7.1	0.531
Left limb strength (kgf)	26.3 ± 8.3	26.3 ± 9.3	28.0 ± 8.3	27.2 ± 10.0	0.400

Data are expressed as mean ± standard deviation

Maximal isometric quadriceps strength in CKF patients did not differ significantly between PBMT doses or compared with the placebo condition in either the right or left limb. Repeated-measures ANOVA showed no significant effect of dose on muscle strength. The effect sizes (Cohen’s *dz*) for comparisons with placebo were small, indicating the absence of clinically meaningful effects, although the p-value for the right limb was close to the significance threshold (*p* = 0.053). Slightly higher mean values were observed with the 90 J dose, without reaching statistical significance (Table 3).

**Table 3:** Values of maximal isometric quadriceps strength, muscle fatigue, and pain under placebo and PBMT dose conditions

Variables	Placebo (*n* = 14)		30 J (*n* = 14)		60 J (*n* = 14)		90 J (*n* = 14)		*p*-value	Cohen’s dz		
	Mean ± SD	95%CI	Mean ± SD	95%CI	Mean ± SD	95%CI	Mean ± SD	95%CI		30 J vs. Placebo	60 J vs. Placebo	90 J vs. Placebo
Quadriceps strength												
Right limb (kgf)	27.7 ± 7.52	(23.4; 32.1)	27.91 ± 7.88	(23.4; 32.5)	25.68 ± 7.81	(21.2; 30.2)	28.24 ± 8.68	(23.2; 33.3)	0.053	0.035	0.501	0.126
Left limb (kgf)	25.8 ± 8.83	(20.7; 30.9)	26.89 ± 8.05	(22.2; 31.5)	26.69 ± 9.09	(21.4; 31.9)	27.39 ± 10.09	(21.6; 33.2)	0.509	0.311	0.207	0.388
Muscle fatigue (Borg scale)												
Pre	1.72 ± 2.55	(0.24; 3.19)	2.36 ± 2.98	(0.64; 4.08)	2.07 ± 2.89	(0.4; 3.74)	2.00 ± 2.35	(0.64; 3.36)	-	-	-	-
Post	1.29 ± 1.98	(0.14; 2.43)	2.57 ± 2.79	(0.96; 4.18)	1.5 ± 1.83	(0.44; 2.56)	1.57 ± 2.82	(−0.06; 3.2)	-	-	-	-
Δ post-pre	−0.43 ± 1.45	(−1.27; 0.41)	0.21 ± 1.97	(−0.92; 1.35)	−0.57 ± 2.32	(−1.91; 0.76)	−0.43 ± 2.28	(−1.74; 0.89)	0.703	0.429	0.046	0.000
Muscle Pain												
Pre	0.72 ± 1.49	(−0.15; 1.58)	1.07 ± 2.43	(−0.33; 2.48)	1.36 ± 2.44	(−0.05; 2.76)	1.71 ± 2.10	(0.16; 3.27)	-	-	-	-
Post	0.79 ± 1.67	(−0.18; 1.75)	1.07 ± 2.37	(−0.3; 2.44)	0.79 ± 1.67	(−0.18; 1.75)	1.64 ± 3.27	(−0.25; 3.53)	-	-	-	-
Δ post-pre	0.07 ± 0.83	(−0.41; 0.55)	0.00 ± 0.39	(−0.23; 0.23)	−0.57 ± 1.5	(−1.44; 0.3)	−0.07 ± 2.62	(−1.58; 1.44)	0.566	0.098	0.462	0.052

Values of maximal isometric quadriceps strength (Kgf), muscle fatigue (Borg scale), and muscle pain measured before (Pre) and after (Post) the strength test under placebo and PBMT dose conditions. Data are presented as mean ± SD and 95% confidence interval (95% CI). Δ represents the mean change between post and pre-exercise values (Post − Pre). P-values represent comparisons between conditions. Effect sizes were calculated using Cohen’s *dz* for comparisons between each PBMT dose and placebo

**Fig. 4:**
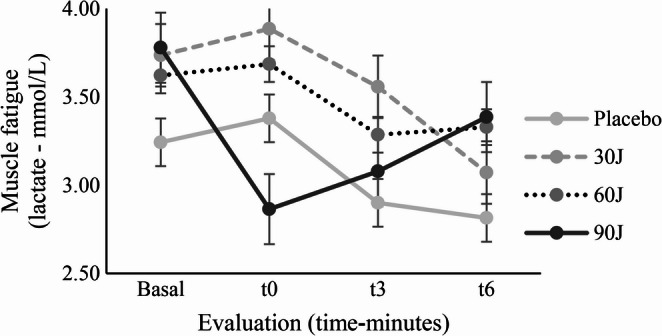
Blood lactate concentrations (mmol/L) measured before PBMT (baseline), immediately after the strength test (t0), and during recovery at 3 and 6 min (t3 and t6)

Muscle fatigue induced by the strength test and assessed by blood lactate concentrations did not differ significantly between PBMT doses at any of the measured time points (baseline, immediately post-exercise, 3 min, and 6 min), as illustrated in Fig. 4 and detailed in Table 4. Repeated-measures ANOVA revealed no significant dose × time interaction (*p* = 0.183; η²*p* = 0.099). Lactate area-under-the-curve (AUC) across the recovery period was also similar between conditions. The mean AUC values were 21.3 mmol·min/L (95% CI: 13.1–29.5) for placebo, 24.9 mmol·min/L (95% CI: 17.3–32.6) for 30 J, 24.0 mmol·min/L (95% CI: 16.3–31.7) for 60 J, and 21.9 mmol·min/L (95% CI: 15.3–28.6) for 90 J, with no statistically significant differences observed between treatments (F = 1.09, *p* = 0.364). Likewise, the perception of leg fatigue assessed by the modified Borg scale was not altered after the application of PBMT, regardless of the dose used (Table 3).

**Table 4:** Blood lactate concentrations used to assess muscle fatigue at baseline, immediately post-exercise, and during recovery following PBMT

Time	Placebo (*n* = 14)		30 J (*n* = 14)		60 J (*n* = 14)		90 J (*n* = 14)		*p*-value		
	Mean ± SD	95%CI	Mean ± SD	95%CI	Mean ± SD	95%CI	Mean ± SD	95%CI	Dose	Time	Dose*Time
Baseline	3.24 ± 2.36	(1.88; 4.6)	3.74 ± 2.1	(2.52; 4.95)	3.62 ± 2.21	(2.35; 4.89)	3.78 ± 2.46	(2.36; 5.2)	0.422	0.100	0.183
t0	3.38 ± 2.21	(2.11; 4.65)	3.89 ± 2.28	(2.57; 5.2)	3.69 ± 1.83	(2.63; 4.74)	2.86 ± 1.79	(1.83; 3.9)			
t3	2.9 ± 2.07	(1.7; 4.1)	3.56 ± 2.01	(2.4; 4.72)	3.29 ± 2.04	(2.11; 4.47)	3.08 ± 1.73	(2.08; 4.08)			
t6	2.81 ± 1.79	(1.78; 3.85)	3.07 ± 1.94	(1.95; 4.19)	3.33 ± 2.08	(2.13; 4.53)	3.39 ± 1.97	(2.25; 4.52)			

Blood lactate concentrations (mmol/L) measured before PBMT (baseline), immediately after the strength test (t0), and during recovery at 3 and 6 min (t3 and t6) under placebo and PBMT dose conditions. Data are presented as mean ± SD and 95% confidence interval (95% CI). P-values represent the effects of dose, time, and dose × time interaction obtained from repeated-measures analysis

Muscle pain was not influenced by the application of any dose of PBMT (Table 3).

## Discussion

This was the first randomized, double-blind, placebo-controlled crossover clinical trial to evaluate the acute effects of different PBMT energy doses (30, 60, and 90 J) on lower limbs in patients with CKF. In this context, no significant acute effects of PBMT were observed on quadriceps muscle strength, fatigue, or pain in patients with CKF undergoing hemodialysis.

Our results showed slightly higher mean values of maximal isometric quadriceps strength when the quadriceps was irradiated with the 90 J/point dose (540 J/leg); however, this difference did not reach statistical significance compared with the other doses. Moreover, the magnitude of this effect was small, and the calculated effect sizes indicated minimal clinical relevance. These findings suggest that, under the conditions tested, PBMT does not produce meaningful acute improvements in quadriceps strength in patients with CKF.

In previous studies with chronic patients, a single application of PBMT was able to increase lower limb muscle strength in patients with chronic obstructive pulmonary disease (COPD) (905 nm, 510 J/leg) [37]. On the other hand, such effects were not observed in patients with heart failure (808 nm, 28 J/leg) [23] or those undergoing coronary artery bypass grafting (CABG) (850 nm, 240 J/leg) [24]. However, a limited number of studies have explored the PBMT effects on muscle strength in CKF patients on HD and there are gaps in the literature regarding its influence on exercise capacity of this group of patients.

Macagnan et al. [19] demonstrated for the first time the acute effect of PBMT during HD. The crossover study with fifteen participants compared different doses of infrared laser irradiation (850 nm) on the finger flexor muscles. The findings suggested that a single session with doses of 60 J and 90 J increased the handgrip strength of these patients, with a superior response at 60 J. Although our parameters align with those defined in the literature [38], the specificity of the target muscles used by Macagnan et al. [19] may have contributed to the positive results. The therapeutic effects of PBMT are associated with the use of appropriate wavelength and energy dose parameters, within a specific therapeutic window [18, 39–41], as well as the suitability of the target tissue or muscle group to be irradiated [35, 41]. Smaller muscles with less adjacent adipose tissue may enhance light penetration into the muscle fibers, optimizing the effects of photobiomodulation [18].

PBMT may produce immediate effects on muscle function through rapid photobiological responses triggered by the absorption of photons by mitochondrial chromophores, particularly CCO [19–21]. This process increases mitochondrial membrane potential, accelerates electron transport in the respiratory chain, and stimulates intracellular ATP synthesis, a mechanism often considered central to explaining the beneficial effects of PBMT on muscle performance [28, 32]. The increase in energy availability appears to be mainly associated with enhanced oxidative metabolism; however, the functional response may vary depending on the muscle group and the metabolic demands of the task [32, 42]. In smaller muscles, such as those of the forearm, commonly assessed through handgrip strength, performance relies heavily on anaerobic metabolism [19].

In this context, the increase in strength observed after PBMT application may be explained by a more efficient integration between aerobic and anaerobic ATP production [28, 32]. In contrast, in larger muscle groups such as the quadriceps, which are involved in tasks requiring high torque production and repeated contractions, the immediate increase in energy availability combined with improved local microcirculation—resulting from nitric oxide photodissociation and subsequent vasodilation—may enhance oxygen and substrate delivery while facilitating the removal of fatigue-related metabolites such as H⁺, ADP, and inorganic phosphate [20, 21, 32]. Additionally, the transient modulation of reactive oxygen species and inflammatory mediators may create a more favorable metabolic environment for muscle contraction [21, 42]. Therefore, these mechanisms may occur shortly after irradiation and help explain the acute effects of PBMT observed in both forearm muscles and the quadriceps, although the magnitude of the response may vary according to muscle size, metabolic characteristics, and the nature of the task evaluated [19, 32, 42].

A critical distinction between the present findings and previous positive studies may relate to the specificity of the target muscle group and the differential penetration of light through subcutaneous tissue. Macagnan et al. [19] irradiated finger flexor muscles, which possess minimal overlying adipose tissue and shallow subcutaneous depth. In contrast, the quadriceps femoris is a substantially larger muscle with potentially greater subcutaneous adiposity in the haemodialysis population, which may attenuate light penetration and reduce optical irradiance reaching the target mitochondria [41, 43]. Given the exponential decay of light intensity with tissue depth, the optical dose delivered to the deepest muscle fibres of the quadriceps may fall below the threshold required for mitochondrial stimulation of CCO [41, 43].

The therapeutic effects of PBMT are associated with the use of appropriate wavelength and energy dose parameters, within a specific therapeutic window [18, 41–45], as well as suitability for the target tissue or muscle group to be irradiated [38, 45]. In a systematic review by Vanin [18], positive results were observed with an energy dose range of 20 to 60 J for small muscle groups and 60 to 300 J for large muscle groups. However, findings in current literature highlight that different PBMT protocols (wavelength, frequency, power, time) can induce different responses in tissues and health conditions, as they do not provide the same dose of treatment [31, 36].

In another randomized clinical trial, Schardong et al. [34] evaluated the chronic effect of PBMT on the quadriceps muscle. CKF patients received 24 treatment sessions (810 nm, 30 J/application site) over 8 weeks, three times/week, during HD. Their findings indicated an increase in the distance covered in the six-minute walk test as well as in the muscle strength of the lower limbs, assessed by the 10-repetition sit-to-stand test for the treated group when compared to the control. Even though we irradiated the same muscle group in our study, the effect of a single application did not yield similar results. The biphasic response relating to the applied dose, described by the Arndt-Schulz Law, can be enhanced by the cumulative effect of the results found in chronic treatment from regular and continuous application of light therapy over time [20, 40].

PBMT has been observed as a potential ergogenic resource for exercise, with the aim of improving performance and recovery after activity [38, 39, 44]. The mechanism of PBMT action provides a reduction in oxidative stress and inflammation in muscles and stimulates the synthesis of muscle proteins [18], as well as promoting the formation of new blood vessels by increasing the supply of oxygen and the total volume of blood around the irradiated area [34]. This may act on processes causing fatigue, such as the accumulation of metabolites and electrolyte imbalance (loss of sodium, potassium, and magnesium through sweat), as well on pain receptors, reducing sensitivity and the transmission of pain signals to the central nervous system [15, 36].

It was hypothesized that PBMT prior to the strength protocol would stimulate muscle metabolism and, consequently, reduce fatigue and pain levels in patients with CKF. However, no effects were found on muscle fatigue measured by Borg scale and blood lactate levels. The analysis of lactate area-under-the-curve (AUC) was performed to evaluate the overall lactate exposure during the recovery period following exercise. The results demonstrated similar AUC values across all experimental conditions, indicating that photobiomodulation did not influence systemic lactate accumulation or its early clearance after the exercise protocol. These findings suggest that the metabolic stress induced by the exercise task and the subsequent recovery kinetics were comparable between sessions. In addition, the relatively small muscle mass involved in the protocol and the moderate metabolic demand of the task may have limited the magnitude of systemic lactate responses, which could partially explain the absence of differences between PBMT doses. These findings corroborate those found in a randomized crossover clinical trial that evaluated the acute effect of PBMT (850 nm, 240 J) on the quadriceps muscle in patients after CABG. Stein et al. [24] did not show any change for functional capacity, fatigue, tissue damage markers and oxidative stress after applying a session of PBMT.

On the other hand, the study by Bublitz et al. [23] evaluated the acute effect of PBMT (808 nm, 28 J) on the quadriceps of patients with heart failure and demonstrated a significant reduction in the perception of effort (Borg scale) for the treated group compared to placebo. Furthermore, the blood lactate level increased for the placebo group, suggesting that PBMT accelerates the removal of peripheral lactate. In this study, the total dose irradiated on the quadriceps femoris was lower when compared to our study, only 4 J/per point. This finding may be related to the effect of the amount of energy absorbed by the cell, generating inhibition or stimulation of intracellular processes [20, 40].

Miranda et al. [34] evaluated the effect of PBMT (superpulsed laser 905 nm; LEDs 875 nm; LEDs 640 nm; 180 J) in patients with chronic obstructive pulmonary disease and found that one application on the quadriceps muscle increased the force of maximal voluntary isometric contraction of the quadriceps and reduced the sensation of dyspnea and fatigue (assessed by the Borg scale). It is important to highlight that, unlike our study, the study by Miranda et al. [37] uses three different wavelengths for irradiation of the lower limb.

Muscle fatigue is a multifactorial phenomenon, and lactate is generally not considered a direct cause of fatigue [46]. However, blood lactate is a well-established physiological marker used to estimate the contribution of the anaerobic metabolism and the metabolic stress associated with exercise intensity [46]. In CKF patients, lactate kinetics may be altered due to metabolic changes and reduced exercise tolerance [6, 9]. Additionally, fatigue in this population is strongly associated with mitochondrial dysfunction, reduced oxidative capacity, muscle atrophy, and other metabolic alterations [6, 7, 9, 11]. Evidence suggests that although resting lactate levels may be slightly higher in patients with end-stage renal disease, peak lactate responses during exercise tend to be lower than in healthy individuals, reflecting a reduced capacity to sustain exercise above the anaerobic threshold [46].

In our study, lactate was not used as an isolated indicator of fatigue but as an objective metabolic marker to complement the subjective assessment of perceived exertion using the Borg scale. Blood lactate concentration was assessed as an indirect marker of metabolic stress and the contribution of anaerobic metabolism during the exercise task [46, 47]. The sequential measurements performed in the present study (baseline, immediately post-exercise, and during recovery at 3 and 6 min) allowed the characterization of the temporal pattern of lactate accumulation and its early clearance following exertion. As expected, lactate concentrations increased immediately after the exercise test and progressively declined during the recovery period, reflecting the physiological process of lactate removal from circulation. When the different PBMT dose conditions were compared, no significant differences were observed in lactate concentrations at any time point. In addition to the analysis of absolute values, exploratory evaluation of the recovery pattern indicated a similar rate of decline in lactate levels across conditions, suggesting comparable lactate clearance dynamics between sessions [42]. Therefore, under the conditions of the present protocol, photobiomodulation did not appear to influence systemic lactate accumulation or the early recovery kinetics following exercise.

Our findings also did not demonstrate any change in pain levels in the lower limbs after the application of PBMT compared to placebo PBMT. This result corroborates that found by Schardong et al. [34] where PBMT, even when applied chronically, had no effect on reducing lower limb pain in patients with CKF undergoing HD. We emphasize that the baseline pain level of the patients evaluated was low, therefore, the lack of results, regardless of the therapeutic dose used, is justified. Although pain is a common symptom reported by 58–84% of patients with CKF [12, 45], this was not a symptom observed in the sample of our study.

The absence of statistically significant effects from a single photobiomodulation session does not necessarily preclude clinical benefits from chronic or repeated PBMT protocols. The findings of Schardong et al. [34], demonstrating functional improvements in the six-minute walk test and sit-to-stand performance following 24 sessions (three times weekly for 8 weeks), suggest that cumulative photobiomodulation effects may emerge only after repeated mitochondrial stimulation and associated adaptive responses. Future investigations should systematically evaluate whether cumulative photobiomodulation sessions (e.g., 2–3 times weekly for 4–8 weeks) delivered prior to structured resistance exercise programs yield functional benefits detectable through both acute functional measures and mechanistic biomarkers. Additionally, investigation of optimal inter-session intervals and combined acute-plus-chronic protocols may identify conditions under which PBMT enhances exercise capacity and muscle performance in CKF populations.

Finally, it is important to clarify that, although the repeated-measures analysis did not demonstrate a statistically significant dose–time interaction, the observed effect size was classified as moderate, suggesting some variability in responses across sessions. This variability may be partially explained by the distribution of treatment sequences. Although 24 sequences were possible, three participants received the same treatment sequence, while the remaining eleven followed different sequences. Importantly, the absence of statistically significant difference between conditions indicates that this variability was not sufficient to support the presence of a clinically relevant residual or carryover effect. Together, these findings suggest that the one-week washout period was adequate to minimize potential carryover between sessions, which is consistent with previous reports in the literature.

This study has limitations that should be considered when interpreting its results. The subjectivity and fragility of the scales used for pain and perceived exertion assessment, despite being validated, may also have influenced the results. In addition, a notable limitation affecting interpretation of the pain-related findings is the low baseline pain experienced by the study sample (mean visual analogue scale 0.72–1.71/10), which contrasts with reported prevalence estimates indicating that 58–84% of patients with chronic kidney failure experience clinically meaningful pain. This discrepancy may reflect the application of exclusion criteria for incapacitating musculoskeletal disease, which was necessary to ensure that participants could safely perform the muscle strength assessments that constituted the primary outcome of the study. Additionally, selection bias toward participants with lower baseline pain or specific characteristics of the recruitment setting may also have contributed to this finding. Consequently, these results regarding pain reduction cannot be generalized to chronic kidney failure populations presenting with clinically significant baseline pain, and future studies should specifically recruit participants with pain levels ≥ 3/10 to adequately investigate the analgesic potential of PBMT.

Another limitation of this study concerns the instrument used for strength assessment. The instrument used (dynamometer by load cell) is a less robust method compared to the gold standard, isokinetic dynamometry. While the load cell is widely used due to its practicality and reliability, it lacks the ability to assess muscle strength at variable speeds and provides less detailed information about muscle performance, such as peak torque and range of motion, which are better captured by isokinetic dynamometry.

For future studies that aim to evaluate the acute effect of PBMT in patients with CKF, it is suggested that higher doses of irradiation be tested prior to the maximal voluntary isometric strength test in the lower limb in this population. In addition, randomized clinical trials that evaluate the chronic effect of the application of PBMT prior to a resistance exercise program are necessary to validate whether patients with CKF can benefit from this therapeutic resource as an adjuvant treatment to improve muscle performance. Finally, it is important to highlight that the PBMT did not result in any adverse effects and was well tolerated by the participants.

## Conclusion

A single application of PBMT did not significantly improve maximal isometric quadriceps strength nor did it alter fatigue or lower limb muscle pain in patients with CKF undergoing hemodialysis, regardless of the dose applied. Although slightly higher strength values were observed with the 90 J/point dose, these differences were not statistically significant. These findings suggest that a single PBMT session, within the tested dose range, does not produce meaningful acute effects on muscle performance or fatigue in this population. Further randomized clinical trials are warranted to investigate whether different irradiation parameters or repeated PBMT sessions may provide therapeutic benefits for patients with chronic kidney failure on hemodialysis.

## Data Availability

All data supporting the findings of this study are available within the paper.
